# When May Our Syphilitics Marry?

**Published:** 1894-08

**Authors:** William S. Gottheil

**Affiliations:** Dermatologist to the Lebanon Hospital, the Northwestern, and the German West Side Dispensaries, New York City; 25 West Fifty-third St.


### Southern Medical Record.

A MONTHLY JOURNAL OF MEDICINE AND SURGERY.

VOL. XXIV. ATLANTA, GA., AUGUST, 1894. No. 8.

### Original Articles.

### WHEN MAY OUR SYPHILITICS MARRY?

BY WILLIAM S. GOTTHEIL, M.D.

Dermatologist to the Lebanon Hospital, the Northwestern, and the German West Side Dispensaries, New York City.

In an able article on the marriage of syphilitics published in the July number of this JOURNAL, Dr. W. G. Porter has laid down some of the rules that should guide us when this inevitable question is presented to us. Nevertheless, all his conclusions would hardly be accepted by the syphilographer of to-day; and would certainly not be received by the laity as their rule of conduct at the dictum of their physician. Herein, to my mind, lies the gist of the whole matter; if we would control the marriages of our syphilitic patients, we must go just as far in the direction of license as the state of medical knowledge and our professional consciences will permit us.

Let us freely admit, to begin with, that a man who has had syphilis is damaged, and for life. He may have had the mildest possible form of the disease; an initial lesion and mild secondary symptoms may have been all its manifestations; and even, as in cases that I have seen, these latter may have been so faintly marked and evanescent that they passed unnoticed. Nevertheless, his tissues are not in the same condition that they were before. They are more vulnerable and less resistant. The harvest of the specific virus has exhausted the soil of some unknown but essential ingredient. Do we not see the same thing after the other contagious exanthemata, to which class, and not to the venereal diseases, syphilis belongs? Have we not all seen cases of scarlet and typhoid, in which the patient recovers, it is true, but with such profound alterations of tissue nutrition that he is never the same man again? Without discussing the relations of tabes and other internal affections to an earlier syphilitic infection, there can be no doubt that a human body that has once undergone syphilis is not the same that it was before; it is more prone to receive the infections that surround it; and less able to resist their effects. Besides all this, it is subject to certain sequelæ of the disease, which themselves not infrequently compromise health and life.

All this, however, has absolutely nothing to do with marriage from a practical standpoint. We are confronted with a social relation, the entrance into which is practically obligatory on every man and every woman; a social relation that is emphasized and rendered necessary by one of the most imperious instincts of our nature. In the vast majority of cases, we may almost say in every case, considerations entirely extra-medical decide whether or not marriage shall take place. Sentimental, esthetic, and social considerations have so preponderating a value that the medical and hygienic factor is almost neglected. Either party may be weak tissued; tuberculosis or insanity may be in either family; past scarlatinas or gonorrheas may have damaged the man or the woman; all these things are of so little account that they hardly enter into the question at all. And it is the same with syphilis. The vast majority of our patients don't ask our permission to marry at all. Feeling well, they consider themselves well, and they just go and marry; and they are right.

Such being the state of affairs, what is the physician's duty in respect to the marriage of his syphilitic patients? It is clearly to restrict his interference to the most unmistakable and absolutely necessary cases; and incidentally to formulate with as much precision as is possible the limits of his bounden duty. The consensus of opinion on this point is practically unanimous. *So long as syphilis is contagious, marriage, save with another past or present syphilitic, is inadmissible.*

Now, the limits of the contagiousness of syphilis have been fairly well ascertained; and we can and are in position to formulate a scheme as follow:

I. Syphilitics may not marry:

1. During the entire primary stage, from the first appearance of the chancre to the entire disappearance of the induration and the inguinal adenopathy. This stage will last at least six months. Secondary symptoms will almost always appear during its continuance, and it will run into the next one. If they do not, a further delay of twelve months is necessary, to await them.
2. During the entire secondary stage, the period of the general eruptions, multiple mucous patches, adenopathies, alopecias, etc. This stage lasts at least twelve months, and is rarely prolonged over two years. But it is not the time so much as the generalized character of the disease manifestations, showing persistent systemic infection and prolonged contagiousness, that marks its limits.

II. Syphilitics may marry:

1. When the disease is in its tertiary stage. This stage lasts from the disappearance of the general symptoms to the end of the patient's life. He may or may not have symptoms of the disease or its sequelæ, but it is no longer contagious, and he cannot communicate it to his partner.
2 When a period of one year has elapsed since the appearance of the last symptom of the disease.

In point of fact, syphilis, at all events during the general and contagious portion of its course, is a self-limiting disease. Treated or untreated it runs its course in a definite though varying and undeterminable time. Treatment shortens the period and prevents the appearance of the tertiary sequelæ. And as all the evidence tends to show that these sequelæ are not contagious, we are justified in giving the patient the benefit of the doubt when they are the only evidences of the disease. To forbid a patient to marry because he has a chronic, scaly, palmar patch, or a tibial node, is to extend our medical authority beyond the justifiable point.

Over and above this, it is useless. Tertiary syphilitics do marry, no matter what the doctor says. And multitudes of persons with maladies far more dangerous to their partners and offspring marry every day. We have all had patients marry who have been distinctly affected with tubercular disease; and I have recently had an instance of a patient marrying into a family every member of which was affected either with exophthalmos, or goitre, or both, one member being in addition congenitally blind and with deformed and atrophied facial bones. To preserve our authority, let us avoid straining it, and let us continue our prohibition of the marriage of syphilitics within the narrowest limits that are possible.

25 *West Fifty-third St., New York City.*

